# *Lycium barbarum* polysaccharide as a retinoprotective agent: A meta-analysis of preclinical evidence from rodent models of retinopathy

**DOI:** 10.4103/NRR.NRR-D-25-00772

**Published:** 2025-12-30

**Authors:** Li Jiang, Erjin Wang, Shengpeng Wang, Yitao Wang

**Affiliations:** 1 State Key Laboratory of Mechanism and Quality Research of Chinese Medicine, Institute of Chinese Medical Sciences, University of Macau, Macao Special Administrative Region, China; 2 Macao Center for Research and Development in Chinese Medicine, Institute of Chinese Medical Sciences, University of Macau, Macao Special Administrative Region, China

**Keywords:** antioxidant, apoptosis, experimental animal models, Lycium, meta-analysis, neuroinflammation, neuroprotection, polysaccharide, retinal diseases

## Abstract

*Lycium barbarum* polysaccharides are considered the primary active ingredient of *Lycium barbarum*, and their therapeutic effects on retinal diseases have been extensively described. However, a systematic review and meta-analysis of these studies have not been conducted previously. This review aims to systematically review and meta-analyze published animal studies to investigate the mechanisms of the antioxidative, anti-inflammatory, anti-apoptotic, and neuroprotective effects of *Lycium barbarum* polysaccharides in rodent models of retinal diseases. To objectively and quantitatively compare the efficacy of *Lycium barbarum* polysaccharides in rodent models of retinal disease, a systematic review was conducted to search the PubMed and Web of Science databases (from inception to July 2024) for studies conducted in animals that met all a priori inclusion criteria. The included 27 studies reported outcomes on retinal structure (outer nuclear layer thickness) or function (electroretinogram b-wave amplitude). The methodological quality, assessed using the SYRCLE bias risk assessment tool, indicated that the overall risk of bias in the included literature was predominantly moderate. The results of the meta-analysis conducted using RevMan 5.4.1 software found that *Lycium barbarum* polysaccharides are protective against retinal injury in animal models, as evidenced by increases in the thickness of the outer nuclear layer and b-wave amplitude. The mechanisms involved include antioxidant effects, anti-inflammation, anti-apoptosis, and modulation of glia-driven neuroinflammation. Findings from this review confirm the protective actions of *Lycium barbarum* polysaccharides on retinal outer nuclear layer thickness and neuronal cells in rodent models of retinal diseases and may help propose strategies for future translational research on *Lycium barbarum* polysaccharides.

## Introduction

Retinal diseases, including glaucoma, diabetic retinopathy (DR), age-related macular degeneration (AMD), retinitis pigmentosa (RP), ischemia/reperfusion (I/R) injury, and traumatic optic neuropathy (TON), are leading causes of irreversible vision loss and are an escalating public health challenge (Tang et al., 2023; Yang et al., 2024; Khan et al., 2025). Their pathogenesis is complex, involving various pathological processes such as oxidative stress, inflammatory responses, vascular abnormalities, and neuronal degeneration (Yau et al., 2012; Tham et al., 2014; Wong et al., 2014). Statistics indicate that approximately 93 million people worldwide suffer from diabetic retinopathy, 196 million from AMD, and 64 million from glaucoma (Mehta and Mehta, 2024). The global burden of these diseases is projected to rise substantially due to aging populations and the increasing prevalence of diabetes, suggesting the urgent need for effective and accessible therapeutic strategies. Beyond the sheer numbers, these conditions impose a significant socioeconomic impact, reducing quality of life and productivity, particularly for working-age adults affected by diabetic retinopathy. Therefore, the development of therapeutic strategies that can effectively halt or slow disease progression has garnered considerable attention from the scientific community.

Glaucoma, currently the second leading cause of global blindness, affects approximately 76 million people, with projections indicating that this number will rise to 111.8 million by 2040 (Kang and Tanna, 2021; Joshi et al., 2022). Although elevated intraocular pressure (IOP) is the principal risk factor, a substantial proportion of patients continue to lose vision despite well-controlled IOP, indicating that IOP reduction alone is insufficient to halt retinal ganglion cell (RGC) apoptosis (Tsai, 2020; Hu et al., 2025; Sato et al., 2025). This phenomenon highlights the critical importance of developing IOP-independent neuroprotective therapies that directly target the underlying mechanisms of neuronal degeneration. Diabetic retinopathy is the foremost cause of blindness in working-age individuals, affecting roughly 35% of the 415 million people living with diabetes, with the risk of vision loss rising sharply with disease duration (Sabanayagam et al., 2016; Chen et al., 2025). RP comprises a heterogeneous group of inherited disorders characterized by progressive photoreceptor degeneration, with a worldwide incidence of approximately 1 in 4,000 and no curative therapy currently available (Shastry, 2008; Lee et al., 2025; Rong et al., 2025; Wu et al., 2025). I/R injury constitutes a common pathological process in acute angle-closure glaucoma, retinal vein occlusion, and other ischemic retinopathies, provoking substantial neuronal death and functional impairment (Schmid et al., 2014; Chong et al., 2020; Chronopoulos et al., 2023; Wei et al., 2023). Moreover, acute or chronic retinal ischemia, such as that seen in acute angle-closure glaucoma or central retinal artery occlusion, induces simultaneous loss of photoreceptors and RGCs, for which effective interventions are lacking (Yao et al., 2021; Patel et al., 2023; Wang et al., 2023; Antonetti et al., 2024).

**Highlights**

• The multi-target synergistic mechanism of *Lycium barbarum* polysaccharides.

• Quantification of the protective effects of *Lycium barbarum* polysaccharides on retinal structure and function.

• Confirmation of the broad protective effects of *Lycium barbarum* polysaccharides against glaucoma and diabetic retinopathy.

• Potential development of *Lycium barbarum* polysaccharides as a multi-target retinal protection drug.

Currently, therapeutic modalities are markedly limited. Laser photocoagulation, while effective in ablating neovascular lesions in diabetic retinopathy, simultaneously destroys peripheral retina and constricts the visual field (Distefano et al., 2017; Simmonds et al., 2025). Anti-vascular endothelial growth factor (VEGF) agents suppress aberrant angiogenesis yet fail to address the underlying neurodegeneration (Latifi-Navid et al., 2023; Cammalleri and Bagnoli, 2025). Moreover, their clinical utility is curtailed by the need for repeated intravitreal injections, high costs, and risks of endophthalmitis, vitreous hemorrhage, and fibrosis (Oosthuyse et al., 2001; Cheng et al., 2021; Watson and Al-Samkari, 2021). Additionally, a significant subset of patients exhibits suboptimal responses or develops tolerance to anti-VEGF therapy over time, necessitating alternative or adjunctive treatment options. Vitrectomy and other surgical interventions can manage late-stage complications but carry inherent risks of infection and retinal detachment (Burdová et al., 2023). Neuroprotective compounds, hampered by their inability to reverse pre-existing neuronal loss and by inconsistent efficacy across heterogeneous retinal degenerations, have not achieved routine clinical adoption (Kovács-Valasek et al., 2023; Patel et al., 2023; Oshitari, 2024). The repeated failures in clinical trials suggest that a paradigm shift towards multi-target, pathway-modulating approaches may be necessary to effectively combat the multifactorial nature of retinal degenerative diseases. Therefore, developing new therapies to induce optic nerve regeneration and functional recovery has become a focal point in recent studies.

Natural medicines have shown unique advantages in the field of neuroprotection and regeneration due to their multi-component, multi-target, and holistic regulatory characteristics (Parvez, 2018; Chen et al., 2022; Yao et al., 2023; Li et al., 2024). This “network pharmacology” approach aligns well with the complex pathogenesis of retinal diseases, offering the potential to simultaneously modulate multiple pathological pathways, thereby enhancing efficacy and reducing the likelihood of compensatory mechanisms that can undermine single-target therapies. Traditional Chinese medicine (TCM) has prescribed *Lycium barbarum* (goji berry) for “brightening vision” for more than two millennia (Ali et al., 2025; Wang et al., 2025; Wei et al., 2025). The berry contains approximately 40% polysaccharides by weight, and recent advances in extraction technology have markedly increased the yield and purity of *Lycium barbarum* polysaccharides (LBPs), facilitating both preclinical and clinical investigations. Consequently, modern research has converged on these water-soluble constituents. LBPs are heteropolymers composed of glucose, galactose, rhamnose, arabinose, mannose, and xylose, with a broad molecular-weight distribution. They exhibit high bioavailability and, after oral or intravitreal administration, readily traverse the blood-retinal barrier to achieve therapeutic concentrations in the retina. Crucially, their multi-target, synergistic mechanisms of action distinguish them from conventional single-pathway therapeutics.

LBP can play an antioxidant role through various mechanisms, including scavenging reactive oxygen species (ROS) and related free radicals, upregulating endogenous antioxidant mechanisms such as superoxide dismutase (SOD), glutathione peroxidase (GSH-Px), and catalase (CAT) (Mi et al., 2012a; Qi et al., 2014). It also suppresses oxidative stress signaling pathways (Liang et al., 2021) and protects mitochondrial function (Chan et al., 2007; Wang et al., 2020). LBP exhibits anti-inflammatory properties through the dual modulation of redox-sensitive signaling pathways (Yang et al., 2014; Hu et al., 2021; Masini et al., 2025): (1) inhibiting nuclear factor kappa-B (NF-κB)-associated pro-inflammatory pathways and (2) upregulating nuclear factor erythroid 2-related factor 2 (Nrf2)-associated antioxidant signaling pathways. This coordinated regulation of NF-κB and Nrf2 is particularly advantageous, as it simultaneously dampens destructive inflammatory responses while promoting cytoprotective pathways, creating a more resilient retinal environment. This parallel regulation reduces the expression of pro-inflammatory molecules such as tumor necrosis factor-α (TNF-α), interleukin-1β (IL-1β), and interleukin-6 (IL-6) (Liu et al., 2018), thereby exerting anti-inflammatory activity. LBP also regulates the function of immune cells and cytokine expression (Li et al., 2019), demonstrating an immunomodulatory mechanism. Additionally, LBP dampens apoptotic cell death by regulating major apoptotic regulators, such as Bcl-2 family proteins (Bcl-2/Bax) and the caspase activation cascades. This anti-apoptotic action is crucial for preserving the terminally differentiated and non-regenerative neurons of the retina, directly contributing to the maintenance of visual function. Nevertheless, the heterogeneity of experimental models and inter-laboratory measurement variability have yielded discordant data that cannot yet guide rational clinical dosing. A comprehensive and systematic meta-analysis is, therefore, indispensable to establish the definitive therapeutic efficacy of LBPs across retinal diseases.

The rationale for focusing on rodent models in this synthesis is multifaceted. While acknowledging the anatomical differences between rodent and human eyes, particularly the absence of a macula in rodents, these models provide unparalleled advantages for mechanistic dissection. Rodent models have been extensively utilized in research on retinal diseases and are considered reliable models for exploring disease mechanisms and validating drugs. While non-human primates offer superior macular homology and species such as cats and dogs provide high-acuity visual fields (Winkler et al., 2020; Petersen-Jones and Komáromy, 2024), their prohibitive costs, ethical complexities, and limited genetic tools restrict scalability for mechanistic studies. Rodents overcome these barriers through extensive genetic toolkits (e.g., Cre-lox systems enabling precise spatiotemporal gene manipulation (Fradot et al., 2024), allowing faithful recapitulation of inherited retinopathies via transgenic models. Crucially, their standardized retinal vasculature mirrors human ischemic pathophysiology (Cammalleri and Bagnoli, 2025), facilitating robust investigation of conditions such as diabetic retinopathy using streptozotocin (STZ) induction (Agarwal and Agarwal, 2017). Thus, while tree shrews or pigs may better mimic specific human ocular features, rodents remain indispensable for dissecting molecular pathways and initial therapeutic validation, providing a critical bridge to targeted studies in higher-order models during later translational phases.

The laser-induced AMD model (Lambert et al., 2013), the STZ-induced diabetic retinopathy model (Stitt et al., 2016; Wang et al., 2019), the high intraocular pressure model (Chiu et al., 2010; Mi et al., 2012a), and hereditary retinal degeneration models (rd1 and rd10 mice) (Wang et al., 2014; Liu et al., 2018) allow for the simulation and systematic examination of the therapeutic effects of LBP, with pathological features resembling human retinal diseases (Ambati et al., 2004; Weinreb et al., 2014; Stitt et al., 2016). Despite the increasing number of individual preclinical studies reporting the benefits of LBP in these models, the evidence remains fragmented. Current literature regarding the use of LBP in rodent models of retinal diseases is relatively scarce, and no systematic examination or overall analysis has been conducted. However, the existing literature is constrained by several critical limitations: (1) pronounced model heterogeneity—acute ischemia (60 minutes) *versus* chronic ocular hypertension (12 weeks)—invokes divergent injury mechanisms, yielding marked variability in LBP effect sizes; (2) wide dose dispersion, with reported regimens ranging from 1 to 400 mg/kg, precludes the construction of reliable dose–response curves; and (3) a fragmented mechanistic focus, as most studies investigate single pathways in isolation. This lack of integration makes it difficult to discern the overall strength of the evidence, the consistency of effects across different disease contexts, and the relative importance of various mechanisms of action. Although rodent paradigms remain indispensable for mechanistic dissection, their translational relevance is tempered by an incomplete recapitulation of human disease complexity. Consequently, a rigorous systematic review and meta-analysis to quantitatively integrate outcome metrics across heterogeneous models is scientifically imperative. Therefore, this study seeks to fill this critical knowledge gap by providing the first comprehensive quantitative synthesis of the efficacy of LBP in rodent models of retinal disease.

Although several reviews have summarized the medicinal value of LBPs (Mi et al., 2013; Ni et al., 2021; Zhou et al., 2022; Al-Wraikat et al., 2024), no systematic review or meta-analysis has specifically evaluated their efficacy in retinal diseases. Existing literature primarily focuses on the general bioactive properties of LBPs (e.g., antioxidant and immunomodulatory effects) or their applications in non-ocular conditions (Ni et al., 2021; Qi et al., 2022). While preclinical studies report the retinoprotective potential of LBP across diverse rodent models, these findings remain fragmented and lack a quantitative synthesis. The absence of a comprehensive meta-analysis limits clinical translation by obscuring consensus on efficacy magnitudes, optimal dosing, and mechanistic consistency across disease contexts.

Based on the background provided, this study aims to systematically evaluate the neuroprotective effects of LBP on structural outcomes (e.g., outer nuclear layer [ONL] thickness) and functional outcomes (e.g., *b*-wave amplitude) in rodent models of retinal diseases. It will explore the advantages and disadvantages of different animal models and systematically evaluate research results through meta-analysis, establishing an evidence base to support the potential of LBP as a neuroregenerative agent for retinal disorders. This study will conduct the first meta-analysis of the efficacy of LBP across six rodent models of major retinal diseases (glaucoma, diabetic retinopathy, RP, AMD, I/R injury, TON) and integrate the mechanistic understanding of the multi-target actions (antioxidant, anti-inflammatory, anti-apoptotic, and glial modulation) of LBP to elucidate pathways relevant to neuronal survival and regeneration. We aim to bridge LBP research with neural regeneration by quantitatively synthesizing evidence on LBP-mediated neuroprotection in retinal neurons, particularly photoreceptors and RGCs, whose degeneration underlies irreversible vision loss. This study will confirm the therapeutic potential of LBP, identify key gaps in the current literature, and provide a robust, evidence-based foundation for designing future preclinical and clinical studies aimed at translating this promising natural product into a viable therapy for devastating retinal diseases.

## Methods

All studies retrieved from the databases were evaluated according to the reporting criteria for systematic meta-analysis reviews of animal experiments. Two independent investigators conducted a systematic literature search using predefined keywords, screened the retrieved studies against the inclusion and exclusion criteria, and subsequently performed data extraction, quality assessment, and meta-analysis. This meta-analysis study was conducted in accordance with the Preferred Reporting Items for Systematic Reviews and Meta-Analysis (PRISMA) 2020 Statement (Page et al., 2021).

### Literature search and selection

Articles involving LBP that examined its influence on the optic nerve in rodents were sought through a systematic literature search. The PubMed and Web of Science databases (Core Collection) were utilized, covering papers from the establishment of the databases until July 2024. Our search query was based on “[*Lycium polysaccharide* (Title/Abstract) OR *Lycium barbarum* polysaccharide (Title/Abstract)] AND [retinal (Title/Abstract) OR retina (Title/Abstract)]”.

### Inclusion and exclusion criteria

#### Inclusion criteria

**(1) Study subjects:** All included studies must utilize rodent models of retinal diseases, regardless of rodent species, sex, body weight, or age. Retinal disease models include, but are not limited to, diabetic retinopathy, AMD, RP, and I/R-induced retinal injury models.

**(2) Interventions:** The intervention group must receive LBP for prevention or treatment, without the concurrent administration of other drugs or physical therapies. The concentration of LBP, treatment duration, and administration route (e.g., oral gavage, intraperitoneal injection) must be explicitly stated.

**(3) Control measures:** The control group shall receive either a placebo or sham surgery, with no retinal disease modeling and no LBP intervention.

### (4) Outcome measures:

Result measures: Histological evaluation (thickness of ONL), electronic physiology evaluation (b-wave amplitude), indicators of oxidative stress (ROS, malondialdehyde [MDA], SOD, GSH), indicators of inflammation (TNF-α, NF-κB, glial fibrillary acidic protein [GFAP]), indicators of apoptosis (caspases), and indicators of neurotoxicity (RAGEs, AGEs, Occludin, Glutamate, Homocysteine).

Histological evaluation (thickness of the outer nuclear layer), electrophysiological evaluation (b-wave amplitude), indicators of oxidative stress (ROS, MDA, SOD, GSH), indicators of inflammation (TNF-α, NF-κB, glial fibrillary acidic protein [GFAP]), indicators of apoptosis (caspases), and indicators of neurotoxicity (receptor for advanced glycation end products [RAGEs], advanced glycation end products [AGEs], occludin, glutamate, and homocysteine).

**(5) Study type:** Only randomized controlled animal trials (RCTs) will be included, with the randomization methods (e.g., random number table, computer-generated sequence) explicitly described.

***Exclusion criteria:*** Cell models, non-rodent studies, or clinical research were excluded. Review articles, replicated studies, or abstracts without full texts were also excluded. Additionally, studies that did not evaluate the impact of LBP on retinal disease in rodent models were excluded. Experiments that combined LBP with other chemicals or drugs in studies on retinal disease in rodent models were also excluded.

### Data elicitation

Researchers LJ and EW independently extracted data from the text, figures, and tables of published papers. Any disagreements were resolved through cross-validation and discussion to reach a consensus. When numerical data needed to be extracted from figures, both researchers independently used GetData Graph Digitizer (v2.26) (https://getdata-graph-digitizer.software.informer.com/) to digitize the data points. The absolute discrepancy between values extracted by the two researchers was required to be less than 3%. Values meeting this criterion were averaged and included in the final analysis, along with the following characteristics:

(1) Author and year of publication.

(2) Experimental animals (species, gender, weight, and age).

(3) Retinopathy model and methods.

(4) Administration and period: LBP treatment details (route of administration, treatment duration).

(5) Animal experimental groups and numbers: including normal control group, model group, and different doses of LBP treatment groups.

(6) Outcome measures: including primary outcomes and effect indices related to retinal morphology (electroretinography [ERG]; hematoxylin and eosin staining [H&E]) and secondary outcomes related to neuroprotective mechanisms (immunohistochemistry [IHC]; immunofluorescence [IF]; quantitative real-time polymerase chain reaction [qPCR]; retinal color Doppler flow; reverse transcription-polymerase chain reaction [RT-PCR]; western blotting analysis).

(7) Pharmacological activity/mechanism: experiments based on the possible neuroprotective mechanisms of LBP as indicated in the findings (mainly including anti-oxidative stress, anti-inflammatory effects, modulation of apoptosis, and direct neuroprotective effects).

### Quality assessment

Two reviewers independently used the downloaded RevMan 5.4 software (Cochrane Collaboration, http://ims.cochrane.org/revman/download) to create a risk of bias assessment form and utilized the SYRCLE (Systematic Review Centre for Laboratory Animal Experimentation) tool to evaluate the methodological quality of animal studies (Hooijmans et al., 2014). The SYRCLE bias risk tool is a modified version of the Cochrane bias risk tool, specifically designed for preclinical animal studies. It includes 10 domains of bias: sequence generation, baseline characteristics, allocation concealment, random housing of animals, blinding of caregivers/investigators, random outcome assessment, blinding of outcome assessors, incomplete outcome data, selective outcome reporting, and other sources of bias. The tool categorizes biases into six types: selection bias, performance bias, detection bias, attrition bias, reporting bias, and other potential biases. The risk level is determined by evaluating the 10 domains, which are scored as ‘Yes’ (green, low risk), ‘Not clear’ (yellow, unclear risk), and ‘No’ (red, high risk).

### Outcome measures

#### Study-level quality

Risk of bias was assessed using the SYRCLE tool. Analytical indicators included sample size, randomization method, allocation concealment, blinding, data completeness, and potential publication bias.

#### Treatment-level efficacy

**Primary outcomes:** Retinal structure (outer nuclear layer thickness or cell-layer count) and retinal function (electroretinogram b-wave amplitude).

**Secondary outcomes:** Oxidative stress biomarkers (ROS, MDA, SOD, GSH, and Nrf2/heme oxygenase-1 [HO-1]); inflammatory mediators (tumor necrosis factor-alpha [TNF-α], interleukin-1 beta [IL-1β], interleukin-6 [IL-6], NF-κB, Iba-1, CD68); apoptosis regulators (Bcl-2/Bax ratio, cleaved caspases-3/7/9, poly (ADP-ribose) polymerase [PARP]); and glial reactivity indices (glial fibrillary acidic protein [GFAP], glutamine synthetase [GS], aquaporin-4 [AQP-4]).

### Statistical analysis

We constructed forest plots using RevMan 5.4.1 (Cochrane Community, London, UK) for the relevant results of the included studies. We also used mean difference (*MD*) as the effect measure for continuous variables and used a 95% confidence interval (*CI*) as an effect measure. Statistical significance of differences was defined as a *P*-value of less than 0.05 (*P* < 0.05). We conducted a heterogeneity test for the outcome indicators in the study. If *I*^2^ was less than 50% and *P* was greater than 0.10, indicating no significant heterogeneity among the studies, we used a fixed-effects model for analysis. Conversely, if *I*^2^ was greater than or equal to 50% and *P* was less than or equal to 0.10, indicating substantial heterogeneity among the studies, we used a random effects model for analysis.

## Results

### Search results

**Figure 1:**
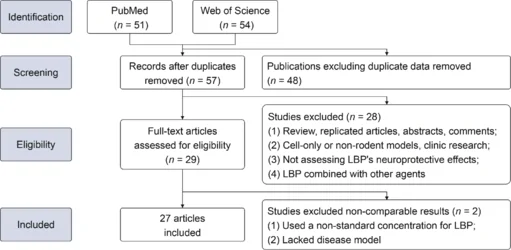
PRISMA flowchart for literature search of randomized animal experiments. PRISMA: Preferred Reporting Items for Systematic Reviews and Meta-Analyses; RCT: randomized controlled trial.

A total of 105 articles were screened by searching the databases Web of Science (54 articles) and PubMed (51 articles). After removing 48 duplicates, 57 articles remained for screening based on title and abstract. Following this, 28 articles were excluded: 11 were reviews, conference, or case reports; 9 were cellular models; 1 was a rabbit model; 2 were clinical studies; 1 was an Alzheimer’s disease model study; 1 had no model group; and 3 lacked full text. Upon reviewing the remaining 29 articles, it was found that one article provided experimental concentrations of LBP based on an unusual mass percent, resulting in information that was inconsistent with the dosage relationships in other papers. Additionally, another article did not propose a disease modeling framework in its experiments, leading to further inconsistencies in the data. Consequently, we discarded these two articles. In total, 27 papers were included in the meta-analysis (**Figure 1**).

### Study characteristics

In **Table 1**, the 27 included studies utilized three rodent species: Sprague–Dawley (SD) rats (*n* = 17, 62.96%), mice (*n* = 7, 25.93%), and transgenic mice (*n* = 3, 11.11%). The distribution of animal sex was as follows: male (*n* = 13), female (*n* = 8), and unspecified (*n* = 6, including all transgenic mouse studies). Five retinal disease models were investigated: glaucoma (*n* = 8, 29.63%), DR (*n* = 5, 18.52%), RP (*n* = 4, 14.81%), I/R injury (*n* = 3, 11.11%), AMD (*n* = 2, 7.41%), and ON (*n* = 5, 18.52%).

**Table 1:** Characteristics of included research articles

Study	Species (gender, weight, age) and model	Retinopathy model and methods	Administration and period	Group	Outcome measures (change with LBP: ↑or↓)	Pharmacological activity/mechanism
He et al., 2014	SD rats (male, 300–350 g, 8 wk)	I/R: raise the IOP to 130 mmHg for 60 min.	i.g., 1 wk.	(1) Sham-operated (*n* = 5); (2) I/R model (*n* = 5); (3) I/R + LBP 1 mg/kg/d (*n* = 5)	(1) RGCs↑, retinal cell apoptosis↓, retinal ganglion cells loss↓, chat-positive cells↑, chat protein levels in the retina↑, ROS↓, Nrf2↑, HO-1↑; (2) chat protein concentrations↑	LBP exerted its neuroprotective effects by activating Nrf2 and increasing HO-1 protein expression.
Wang et al., 2017	SD rats (male, 250 ± 25 g)	DR: injected with STZ.	i.g., 4 wk	(1) Blank control (*n* = 5); (2) DM model (*n* = 6); (3) DM + 6% LBP, 0.5 mL/d (*n* = 7)	MDA↓, SOD↑, VEGF↓	LBP reduced the damage to mitochondria, prevented nerve cell death and stopped vascular lesions from developing through its antioxidant effect.
Yang et al., 2017	C57BL/6 mice (male, 10–12 wk)	I/R: subjected to 2 h of ischemia.	i.g., 2 wk (started 7 d before ICA)	(1) Sham-operated (*n* = 6); (2) PBS (*n* = 6); (3) 1 mg/kg/d (*n* = 6); (4) 10 mg/kg/d (*n* = 6)	(1) ERG: a-wave, b-wave↑, oscillatory potentials (ops)↑; (2) IRL↑, calretinin↑, PKC-α↑, calretinin↑, GFAP↓	LBP ameliorated ischemia-associated retinal functional impairment, concurrently attenuating neuronal apoptosis and reactive gliosis.
Pan et al., 2019	SD rats (male, 250 ± 20 g)	DR: injected with STZ.	i.p., 12 wk	(1) Normal control (*n* = 6); (2) DM model (*n* = 6); (3) DM + LBP 1 mg/kg/d (*n* = 6)	RGCs↑, ROS↓, Nrf2↑, HO-1↑	In diabetic rodent models, LBP demonstrated potent antioxidant properties by activating the Nrf2/HO-1 pathway, effectively mitigating oxidative damage and preserving retinal neurons.
Chan et al., 2007	SD rats (female, 250–280 g, 10–12 wk)	Glaucoma: induced OH by argon laser (0/7 d).	i.g., 3 or 5 wk (started 1 wk before first argon laser)	(1) PBS control (*n* = 7); (2) LBP 1 mg/kg (*n* = 7); (3) OH + PBS (*n* = 6); (4) OH + LBP 1 mg/kg (*n* = 5)	Percentage of RGC loss (%) ↓	LBP demonstrated reduction in the loss of RGCs, while IOP remained unaltered and the neuroprotective effects persisted for 4 wk.
Chiu et al., 2009	SD rats (female, 250–280 g)	Glaucoma: induced OH by argon laser (0/7 d).	i.g., 5 wk (started 1 wk before OH)	(1) Normal control (*n* = 6); (2) OH + PBS (*n* = 6); (3) OH + LBP 1 mg/kg (*n* = 6); (4) OH + LBP 10 mg/kg (*n* = 6); (5) OH + LBP 100 mg/kg (*n* = 6); (6) OH + LBP 1000 mg/kg (*n* = 6)	Microglia marker↑, RGCs↓	LBP modulated microglial activation to exert neuroprotective effects.
Chiu et al., 2010	SD rats (female, 250–280 g)	Glaucoma: induced OH by argon laser (0/7 d).	i.g., 3 wk or 9 d (started 1 wk before OH)	(1) Normal control (*n* = 12); (2) PBS control (*n* = 6); (3) LBP 1 mg/kg (*n* = 6); (4) OH + PBS, 2 d (*n* = 6); (5) OH + LBP 1 mg/kg, 2 d (*n* = 6) (6) OH + PBS, 14 d (*n* = 12) (7) OH + LBP 1 mg/kg, 14 d (*n* = 12)	β B2-crystallin↑	LBP may up-regulate neuronal survival signal β B2-crystallin.
Li et al., 2011	C57BL/6N mice (male, 10–12 wk)	I/R: induced by ligating the right common carotid artery and right ECA for 2 h, then reperfusion for 22 h.	i.g., 8 wk (started 1 wk before I/R)	(1) Normal control (*n* = 7); (2) I/R model (*n* = 7); (3) I/R + LBP 1 mg/kg/d (*n* = 7)	Pyknotic viable cell↑, retinal thickness in the central retina↓, IGG extravasations↓, PAR↓, TUNEL↓, PKC-α↑, Calretinin↑, nNOS↑, GFAP↓, AQP4↓.	LBP protected the retina from I/R injury by reducing apoptosis, inhibiting glial cell activation, decreasing oxidative stress, protecting the blood-retinal barrier integrity, and reducing retinal edema.
Mi et al., 2012a	C57BL/6N mice (male, 20–25 g, 10 to 12 wk)	Glaucoma: induced AOH by inserting anterior chamber with balanced salt solution (1 h, 90 mmHg).	i.g., 11 and 14 d (started 1 wk before AOH)	(1) Non-AOH control (*n* = 7); (2) AOH + PBS control (*n* = 7); (3) AOH + LBP 1 mg/kg/d (*n* = 7)	IRL↑, blood–retinal barrier leakage ↓, AGE↓, RGCs↑, Occludin↑, PECAM-1↑, NG2↑, retinal blood vessel density↑, ET-1↓, RAGE↓, Aβ1–42↓, RAGE↓	LBP exerts vasculoprotective effects via down-regulating RAGE, ET-1, Ab and AGE in the retina. thereby preventing RGCs damage under pathological conditions.
Mi et al., 2012b	SD rats (female, 250–280 g)	Glaucoma: induced by argon laser for at least 2 mon.	i.g., 3 wk (started 1 wk before photocoagulation)	(1) COH + PBS (*n* = 3); (2) COH + LBP 1 mg/kg (*n* = 3)	ET-1↓, ETA↓ and ETB↑.	LBP exerted retinal neuroprotection by inhibiting the ET-1/ETA signaling pathway while activating the ET-1/ETB pathway.
Li et al., 2013	SD rats (female, 250–280 g, 10–12 wk)	PONT: transection of the temporal side of rat optic nerves.	i.g., 2 and 5 wk (started 1 wk before PONT).	(1) PONT-1 wk +PBS (*n* = 7); (2) PONT-1 wk + LBP (*n* = 4); (3) PONT-4 wk + PBS (*n* = 9); (4) PONT-4 wk + LBP (*n*=10)	Fluoro-Gold in the superior retina and inferior retinas↑, mnSOD↑, p-JNK2/3↓, p-c-JUN↓, BDNF, IGF-1↑	LBP delayed secondary RGC degeneration in retinal injury models by inhibiting oxidative stress and the JNK/c-jun pathway.
Chu et al., 2013	SD rats (250–280 g, 12 wk)	PONT: transection of the dorsal part of the optic nerve.	i.g., 5 wk (started 1 wk before PONT)	(1) Normal control (*n* = 5); (2) PONT (*n* = 5); (3) PONT + LBP 1 mg/kg/d (*n* = 5); (4) PONT+PBS (*n* = 5); (5) LBP (*n* = 5); (6) PBS (*n* = 5)	mfERG: N1↑, P1↑, PhNR↑	LBP altered the functional reduction by regulating the signal from the outer retina.
Wang et al., 2014	Rd10 mice RP model	Transgenic mice.	i.g., 12/16/28 d (started from p14 and continued to P25, P29, P41)	(1) WT control mice (*n* = 9); (2) rd10 + LBP 1 mg/kg/d (*n* = 10); (3) rd10 + PBS (*n* = 15)	(1) ONL thickness↓; (2) ERG: a/b wave amplitudes↑, a/b-waves Latency↓; (3) Visual acuity↑, OS/IS length↓, cone density↓, CD68↓, phospho- IκBα (Ser32)↓, TNF-α↓, IL-6β↓, p-IκBα/Total IκBα↓, p65↓, GSH/GSSG↑, HIF-1α↓, caspase-3/7↓, Bax↓, CCL2↓	LBP modulated inflammation and apoptosis in part by inhibiting the expression of NF-κB and HIF-1α.
Li et al., 2015	SD rats (female, 250–280 g, 10–12 wk)	PONT: transection of partial cut of the central nerves.	i.g.,5 wk (started 1 wk before PONT).	(1) Normal control (*n* = 4); (2) PONT + PBS (*n* = 4); (3) PONT + LBP 1 mg/kg/d (*n* = 4)	(1) Axons densities↑, g-ratio↑, myelin thickness; (2) Iba-1-positive labeling areas↓	LBP could inhibit the activation of microglia/macrophages to delay secondary degeneration of the axons.
Zhu et al., 2016	SD rats (male, 200–220 g, 7–8 wk)	RP: injected with MNU.	i.g., 8 or 14 d (started 7 d before injecting MNU)	(1) Normal control (*n* = 4); (2) MNU model (*n* = 4); (3) MNU + LBP 100 mg/kg (*n* = 4); (4) MNU + LBP200 mg/kg (*n* = 4); (5) MNU + LBP400 mg/kg (*n* = 4); (6) LBP 400 mg/kg (*n* = 4)	(1) Total and outer retinal thickness↑; (2) TUNEL-positive apoptotic cell ratio↓; (3) Cleaved caspase protein-9/3/7↓, cleaved PARP expression↑	LBP inhibited MNU-induced apoptosis in rat photoreceptor cells. It protected retinal structure by regulating the expression of PARP and caspase.
Yao et al., 2018	SD rats (male, 250 ± 20 g)	DR: injected with STZ.	i.g., 20 wk	(1) Normal control (*n* = 15); (2) DM, diabetic control (*n* = 5); (3) DM + LBP 200 mg/kg/d (*n* = 5); (4) DM + LBP 400 mg/kg/d (*n* = 5)	(1) ERG: amplitude of a-wave, b-wave↑, and ops↑; (2) Velocity of the PSV↑, EDV (400)↑, CRV, MV (400)↑; (3) ONL↑; (4) The basement membrane↑, GFAP↓, VEGF↓, and PEGF↑	LBP maintained the structural and functional integrity of the retina in DR, probably by modulating the VEGF/PEDF balance.
Liu et al., 2018	Rd1 mice RP model	Transgenic mice.	i.p., once per day, started at postnatal day 4 (P4) lasting until P24	(1) WT mice (*n* = 3); (2) Rd1+ PBS (*n* = 3); (3) Rd1 + LBP 10 mg/kg/d (*n* = 3)	(1) ERG: a wave-dark adapted↓, b wave-dark adapted↑, b wave-photopic↑; (2) photoreceptor number (p14\20\24) ↑ CTBP2↑, cone arrestin, PKCα↑, RGCs↑	LBP improved retinal morphology and function in RD1 mice and delayed RGC loss during photoreceptor degeneration.
Tang et al., 2018	BALB/cJ mice (2–3 mon)	AMD: exposed to a fluorescent light source (5000 lx for 24 h) following 24 h dark adaptation.	i.g., 1 wk	(1) Control [light (-)] (*n* = 4); (2) PBS-treated [light (+)] (*n* = 4); (3) AMD + LBP 130 mg/kg (*n* = 4); (4) AMD + LBP 300 mg/kg (*n* = 4)	(1) ERG: a/b-waves amplitude↑, a/b-wave latency↓; (2) Number of rows of photoreceptor nuclei of the ONL↑; (3) Rhodopsin expression↑, ROS↓, Nrf2↑, TrxR1↑, PARP14↓	LBP up-regulated the antioxidant genes Nrf2 and TrxR1 to protect the cell in the retina from damage caused by light and reduce the level of PARP14 mRNA to delay damage to photoreceptors.
Wang et al., 2019	Rats (180–220 g, 8–12 wk)	DR: injected with STZ.	i.g., 12 wk (start as DM induced)	(1) Control (*n* = 36); (2) DM model (*n* = 36); (3) DM + LBP 250 mg/kg/d (*n* = 36)	(1) EB infiltration capacity↓; (2) thickness of BRB↑, VEGF↓, P-occludin↑, ROCK1↓, P-MLC↓	LBP was found to exert protective effects on the blood-retinal barrier by regulating the Rho/ROCK signaling pathway in diabetic rats.
Lakshmanan et al., 2019a	SD rats (male, 180–200 g, 10 wk)	Glaucoma: induced OHT by inserting anterior chamber with balanced salt solution (80 mm Hg, 120 min).	i.g., 35 d (started 7 d before OHT) or 28 d (started 6 h after OHT)	(1) Sham control (*n* = 5); (2) OHT-vehicle control (*n* = 8); (3) OHT + LBP pretreatment (*n* = 8); (4) OHT + LBP posttreatment (*n* = 8)	(1) Optical coherence tomography: thickness of RNFL↑, IRL↑, ORL↑; (2) ERG: amplitudes of pSTR↑, a/b-wave↑	LBP arrested the secondary degeneration and improved the retinal function.
Lakshmanan et al., 2019b	SD rats (female, 180–210 g, 10 wk)	Glaucoma: induced by tightening Circumlimbal Suture Model (70 mmHg, 15 d).	i.g., 15 wk (start 1 wk before AOH)	(1) Sham control (*n* = 6); (2) AOH vehicle control (*n* = 9); (3) AOH pretreatment + LBP 1 mg/kg (*n* = 8); (4) AOH pretreatment + LBP 10 mg/kg (*n* = 13); (5) AOH posttreatment + LBP 10 mg/kg (*n* = 8)	(1) Retinal layer thicknesses of TRT↑, RNFL, IRL↑, ORL; (2) ERG↑, a/b-wave↑; (3) GCL density ↑, axonal bundle arrangement ↑, β-III-tubulin↑	LBP protects the inner and outer retinal structures and functions before and after nerve injury. It prevents neuronal degeneration after injury and protects RGCs and retinal function.
Li et al., 2019	SD rats (female, 220–240 g, 9–10 wk)	PONT: transection of the temporal side of rat optic nerves.	i.g., 2 and 5 wk (started 1 wk before PONT)	(1) PBS control (*n* = 6); (2) PONT model (*n* = 6); (3) PONT + LBP 1 mg/kg/d (*n* = 6)	(1) RGCs↑; (2) CD68↑, Arginase-1↑.	LBP delayed secondary degeneration of RGCs 4 wk after PONT.
Yao et al., 2020	SD rats (male, 240 ± 20 g, 8 wk)	DR: injected with STZ.	i.p., 8weeks	(1) NC, normal control group (*n* = 10); (2) NC + LBP 50 mg/kg (*n* = 10); (3) DM, diabetic control (*n* = 10); (4) DM + LBP 25 mg/kg (*n* = 10); (5) DM + LBP 50 mg/kg (*n* = 10)	(1) ERG: b-wave amplitude↑; (2) p53↓, active-caspase-3↓, FOXO1↓, BAX↓ and p27kip1↓, SIRT1↑, Bcl-2↑	LBP activated SIRT1 to regulate diabetic cataract.
Mi et al., 2020	C57BL/6N mice (male, 20–25 g, 10–12 wk)	Glaucoma: induced AOH by inserting anterior chamber with balanced salt solution (1 h, 90 mmHg)	i.g.,11 d (started 7 d before AOH)	(1) Non-AOH control (*n* = 7); (2) AOH + PBS control (*n* = 7); (3) AOH + LBP 1 mg/kg/d (*n* = 7)	(1) Astrocytes↓, Müller cells↓, glial cells↓, RAGE↓; (2) GS↓, AQP-4↓, Iba-1↓, APP↓	LBP protected the retina by regulating glial cell activity, targeting astrocyte remodeling for vascular health, and blocking RAGE to prevent damage from injury or disease.
Au et al., 2022	C57BL/6 mice (male, 8–12 wk)	PNI: induced by optic nerve crush (ONCT).	p.o., and i.v., 21 d (p.o., started 1 wk before ONCT and twice injections (i.v., at d 0 and 7 after ONCT)	(1) Normal control (*n* = 5–6); (2) ONCT+ LBP 100 mg/kg (*n* = 5–6)	(1) CTB-labeled regenerating RGC axons↑; (2) RGCs↑, Cholera toxin subunit B↑, RBPMS↑	LBP enhanced the regenerative capacity of neurons, promoting the survival of RGCs and the regrowth of their axons following optic nerve crush or peripheral nerve injury.
Yang et al., 2023	Rd10 mice AMD model (4 wk)	Transgenic mice.	p.o., 28 d	(1) Normal control (*n* = 8); (2) Rd10 model (*n* = 8); (3) Rd10 + LBP (3) 6 g/kg/d (*n* = 8); (4) Rd10 + LBP 7.2 g/kg/d (*n* = 8)	(1) RPE layers↑, ONL thickness↑, RPE↑; (2) ERG: a/b wave amplitude ↑; (3) Bcl-2↑, Beclin1↓, LC3B↓, Atg5↓	LBP regulated the expression of microRNA-181 and Bcl-2, decreasing autophagy and oxidative stress in the retinal pigment epithelium.
Kong et al., 2024	C57BL/6J mice (male, 21 g, 7 wk)	RP: injected with MNU.	i.g., 2 wk (started 7 d before injecting MNU)	(1) Normal control (*n* = 46); (2) MNU model (*n* = 47); (3) MNU+ LbGP 100 mg/kg/d (*n* = 47)	(1) Time in black box↑, visual activity↑; (2) ERG: a/b wave amplitude ↑; (3) ONL layers↑, ONL thickness↑, OS length of the cones↑, the OS thickness of the RODs↑, GFAP, Iba-1↓, CD68↓, IL-6↓, IL-1β↓, NF-κB↓, CD40, iNOS, HIF-1↓	LBP inhibited the cell apoptosis in rat eyes induced by MNU and protected the retina structure by regulating the expression of PARP and caspase.

Aβ_1–42_: Amyloid-beta 1–42; AGE: advanced glycation end product; AMD: age-related macular degeneration; AOH: acute ocular hypertension; AQP4: aquaporin-4; BRB: blood–retinal barrier; CCL2: C-C motif chemokine ligand 2; CCA: common carotid artery; CCL2: C–C motif chemokine ligand 2; CRV: central retinal vein; CTB: cholera toxin B subunit (*n*euroanatomical tracer); CTBP2: C-terminal binding protein 2; DM: diabetes mellitus; DR: diabetic retinopathy; EB: Evans blue; ECA: endothelial cell adhesion; EDV: enddiastolic velocity; ERG: electroretinography; ET-1: endothelin-1; ETA: endothelin A; ETB: endothelin B; GCL: ganglion cell layer; GFAP: glial fibrillary acidic protein; GSH: glutathione; GSSG: oxidized glutathione; HIF-1α: hypoxia-inducible factor 1-alpha; HO-1: heme oxygenase-1; GFAP: glial fibrillary acidic protein; i.g.: intragastric (administration); I/R: ischemia/reperfusion; ICA: internal carotid artery; IgG: immunoglobulin G; IL1β: interleukin1 beta; IL6β: interleukin6; IOP: intraocular pressure; IRL: inner retinal layer; LBP: *Lycium barbarum* polysaccharides; MDA: malondialdehyde; mfERG: multifocal electroretinography; MNU: N-methyl- N-nitrosourea; MV: microvessel; NFκB: nuclear factor kappalightchainenhancer of activated B cells; nNOS: neuronal nitric oxide synthase; Nrf2: nuclear factor erythroid 2-related factor 2; OHT: ocular hypertension; OH: hydroxyl radical; ONL: outer nuclear layer; ORL: outer retinal layer; OS: outer segment (of photoreceptors); OS/IS: outer segment / inner segment (photoreceptor layers); PAR: poly(ADP-ribose); PARP: poly (ADPribose) polymerase; PBS: phosphatebuffered saline; PECAM1: PECAM-1: platelet endothelial cell adhesion molecule-1; PKCα: protein kinase Calpha; PMLC: phosphorylated myosin light chain; PNI: peripheral nerve injury; PONT: partial optic nerve transection; PSV: peak systolic velocity; pSTR: positive scotopic threshold response; RAGE: receptor for advanced glycation end products; RGC: retinal ganglion cell; RNFL: retinal nerve fiber layer; ROCK: Rhoassociated protein kinase; RODs: rod photoreceptors; ROS: reactive oxygen species; RP: retinitis pigmentosa; RPE: retinal pigment epithelium; SD: Sprague-Dawley; SOD: superoxide dismutase; TNFα: tumor necrosis factoralpha; TRT: total retina; TUNEL: terminal deoxynucleotidyl transferase dUTP nick end labeling; VEGF: vascular endothelial growth factor; PEDF: pigment epitheliumderived factor; WT: wild type.

Four primary induction methods were used:

Chemically induced: STZ for all DR models (*n* = 5); N-methyl-N-nitrosourea (MNU) for RP models (*n* = 2).

Surgically induced: Partial optic nerve transection for TON (*n* = 4); optic nerve crush for TON (*n* = 1); microload perfusion for glaucoma (*n* = 3); I/R injury (*n* = 3); argon laser photocoagulation for glaucoma (*n* = 4); tight circumlimbal suture for glaucoma (*n* = 1).

Light-induced: Light exposure for glaucoma (*n* = 1) and AMD (*n* = 1).

Genetic models: Transgenic mice for RP (rd1/rd10 mice, *n* = 2); AMD (rd10 mice, *n* = 1).

All studies utilized LBP as the intervention. Administration routes included oral gavage (*n* = 23, e.g., He et al., 2014; Wang et al., 2017), intraperitoneal injection (*n* = 2, e.g., Liu et al., 2018; Pan et al., 2019), and oral delivery (*n* = 2, e.g., Au et al., 2022), with one study combining oral and intravenous administration (Au et al., 2022). Dosages ranged from 1 to 300 mg/kg. The timing of administration included preventive (administered prior to disease induction, e.g., Chan et al., 2007; Yang et al., 2017) and therapeutic (administered post-modeling, e.g., He et al., 2014; Wang et al., 2017). Treatment periods ranged from 1 week (shortest, He et al., 2014) to 20 weeks (longest, Yao et al., 2018).

### Risk of bias assessment

**Figure 2:**
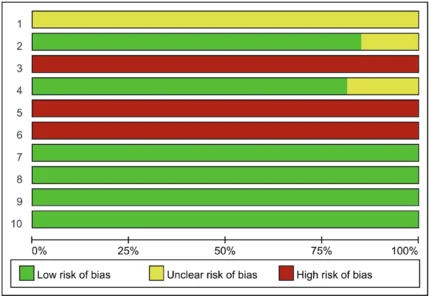
The SYRCLE risk of bias assessment criteria for included research articles. The SYRCLE risk of bias assessment criteria for the included research articles are presented below. Green, yellow, and red colors signify low risk of bias, unclear risk of bias, and high risk of bias, respectively. This figure was generated using RevMan 5.4 software (Cochrane Collaboration, http://ims.cochrane.org/revman/download). 1: Adequacy of allocation sequence generation and application (selection bias); 2: Baseline comparability across groups (selection bias); 3: Adequacy of allocation concealment (selection bias); 4: Random housing of animals during experimentation (performance bias); 5: Blinding of caregivers/investigators (performance bias); 6: Random selection of animals for outcome assessment (detection bias); 7: Blinding of outcome assessors (detection bias); 8: Complete reporting and justification of incomplete outcome data (attrition bias); 9: Freedom from selective outcome reporting (reporting bias); 10: Absence of other sources of bias (other potential biases).

All 27 included studies were randomized controlled trials. While all studies described random group allocation, none detailed the randomization methods (e.g., computer-generated sequences or random number tables), resulting in an ‘unclear risk’ rating for sequence generation (domain 1). In 23 studies (92.6%), baseline characteristics (domain 2) were balanced between the experimental and control groups, including age, sex, and body weight. However, the baseline balances were not described in four studies (Wang et al., 2014; Liu et al., 2018; Tang et al., 2018; Yang et al., 2023). Five studies did not mention the environment in which the animals were kept during the experiment (domain 4) (He et al., 2014; Wang et al., 2014; Pan et al., 2019; Yao et al., 2020; Au et al., 2022). High-risk biases were identified in domains 3, 5, and 6. No study reported on allocation concealment (domain 3), the blinding of researchers/caregivers (domain 5), or random selection for outcome assessment (domain 6). The overall risk of bias profile is summarized in **Figure 2**.

### Assessment of the role of *Lycium barbarum* polysaccharides in retinal disease

#### Lycium barbarum polysaccharides treatment increases the thickness of outer nuclear layer

**Figure 3:**
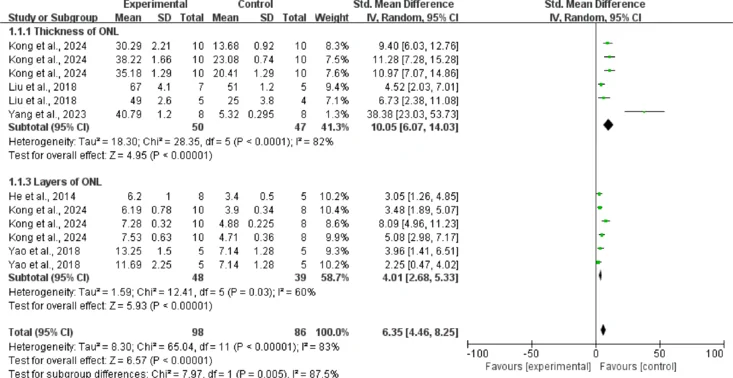
Forest plot for comparison: outer nuclear layer thickness. The forest plot illustrates that the *Lycium barbarum* polysaccharides intervention consistently increased the thickness of outer nuclear layer and the number of cell layers. This figure was created using RevMan 5.4 software (Cochrane Collaboration, http://ims.cochrane.org/revman/download).

Quantitative assessment of the ONL, the photoreceptor-containing stratum, is commonly performed by either measuring ONL thickness or counting the number of ONL cell layers. In this study, we extracted both metrics from eligible articles; the results are depicted in **Figure 3**. Five studies reported ONL outcomes after LBP treatment: three studies (six independent groups) employed ONL thickness measurements (Liu et al., 2018; Yang et al., 2023; Kong et al., 2024), whereas another three studies (six independent groups) reported ONL cell-layer counts (Wang et al., 2014; Yao et al., 2018; Kong et al., 2024). A meta-analysis of data from these five studies (comprising twelve independent experimental groups) revealed that LBP intervention consistently and significantly increased ONL metrics compared to controls (*P* < 0.00001). This effect was observed uniformly regardless of the measurement method used, demonstrating a deterministic protective effect of LBP on photoreceptor survival.

Notably, subgroup analysis indicated higher heterogeneity among studies employing ONL thickness measurements (*I*^2^ = 82%) compared with those using ONL cell-layer counts (*I*^2^ = 60%). This heterogeneity likely stems from several sources: (1) divergent disease induction methods across studies (e.g., genetic models *vs*. chemical-induced models); (2) variations in experimental protocols and measurement techniques between different laboratories; and (3) the inclusion of multiple treatment/control arms within individual studies. Importantly, a leave-one-out sensitivity analysis confirmed that no single study exerted undue influence on the overall effect size, though the limited number of available studies (*n* = 5) may inherently contribute to substantial variability in heterogeneity estimates. The consistently lower heterogeneity observed with layer counting suggests this method may provide more stable and reliable estimates, possibly due to reduced susceptibility to technical variations in tissue processing and measurement. Moving forward, to enhance cross-study comparability and minimize analytical bias, we strongly recommend that future investigations: (1) employ multiple standardized metrics for ONL assessment concurrently and (2) adhere to consistent anatomical locations and time points for quantification. Such standardization will be crucial for generating more homogeneous data pools for future meta-analyses while providing a more comprehensive assessment of the therapeutic effects of LBP.

#### Lycium barbarum polysaccharides treatment increased the b-wave amplitude

**Figure 4:**
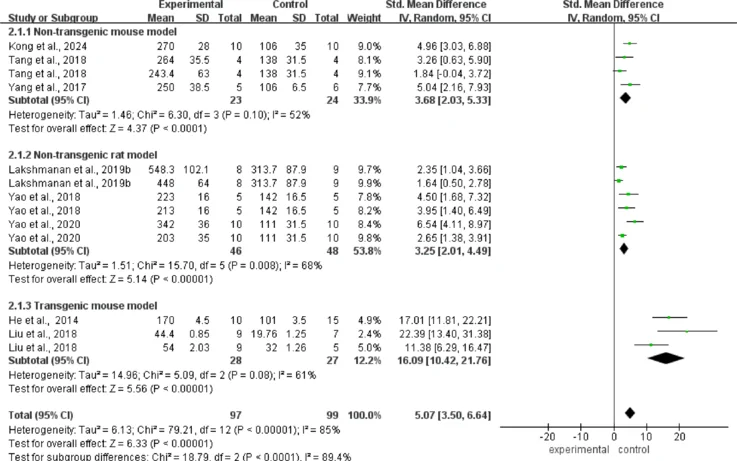
Forest plot for comparison: *b*-wave amplitude. *Lycium barbarum* polysaccharides treatment restored the disease-induced reduction in b-wave amplitude. The magnitude of improvement was most pronounced in transgenic mice. This figure was created using RevMan 5.4 software (Cochrane Collaboration, http://ims.cochrane.org/revman/download.).

As shown in **Figure 4**, a total of six studies with seven different groups were included in the meta-analysis (Yang et al., 2017; Tang et al., 2018; Yao et al., 2018, 2020; Lakshmanan et al., 2019a; Kong et al., 2024). The meta-analysis demonstrated that LBP treatment robustly restored the disease-induced reduction in b-wave amplitude (*P* < 0.00001), confirming a consistent and deterministic beneficial effect of LBP on retinal function. Considerable heterogeneity was observed across the studies (*I*^2^ = 85%). To address this, we performed a subgroup analysis stratified by species and model type, which revealed that the heterogeneity was substantially reduced to a moderate level within each subgroup (non-transgenic mice, *I*^2^ = 52%; non-transgenic rats, *I*^2^ = 68%; and transgenic mice, *I*^2^ = 61%). Notably, the magnitude of improvement was most pronounced in transgenic mice.

The high overall heterogeneity primarily stems from the substantial methodological diversity in disease induction methods across the included studies. The subgroup analysis effectively identified the source of the heterogeneity, indicating that the animal model used is a key contributing factor. The superior effect size observed in transgenic models suggests that LBP may have particular therapeutic advantages for specific disease etiologies, such as inherited retinal degenerations. A leave-one-out sensitivity analysis (results not shown) indicated that no single study disproportionately influenced the overall pooled estimate, reinforcing the stability of our primary conclusion. However, the limited number of studies available for each subgroup remains a constraint, likely contributing to the residual heterogeneity. Future research should aim to standardize electrophysiological assessment protocols, including stimulus parameters and animal age at testing, to minimize technical variability. Additionally, prioritizing studies in well-characterized genetic models could help clarify the specific contexts in which LBP exerts its most potent functional benefits, thereby facilitating its targeted clinical translation.

### Mechanisms of *Lycium barbarum* polysaccharides in retinal disease prevention and treatment

**Figure 5:**
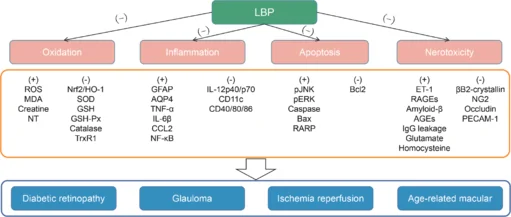
Neuroprotective mechanisms of LBP. LBP exerts a protective effect against retinal diseases through its antioxidant, anti-inflammatory, anti-apoptotic, and neuroprotective properties. AQP4: Aquaporin-4; AGEs: advanced glycation end products; Bax: Bcl-2-associated X protein; BB2-crystallin: beta-2-brystallin; Bcl2: B-cell lymphoma 2; caspase: cysteinyl aspartate specific proteinase; CD11c: cluster of differentiation 11c; CD40/80/86: cluster of differentiation 40/80/86; CCL2: chemokine (C–C motif) ligand 2; Cr: creatine; ET-1: endothelin-1; GFAP: glial fibrillary acidic protein; GSH: glutathione; GSH-Px: glutathione peroxidase; HO-1: heme oxygenase-1; IgG leakage: immunoglobulin G leakage; IL-12p40/p70: interleukin-12 p40/p70; IL-68: interleukin-68; LBP: *Lycium barbarum* polysaccharides; MDA: malondialdehyde; NF-κB: nuclear factor kappa-light-chain-enhancer of activated B cells; NG2: Nogo-2; NT: neuronal thread protein; PECAM-1: platelet endothelial cell adhesion molecule-1; pERK: phospho-extracellular signal-regulated kinase; pJNK: phospho-c-Jun N-terminal kinase; RAGEs: receptors for advanced glycation end products; RARP: receptor-interacting serine-threonine kinase; ROS: reactive oxygen species; SOD: superoxide dismutase; TNF-α: tumor necrosis factor-alpha.

As summarized in **Figure 5**, the 27 included studies collectively demonstrate that LBP has protective effects against retinal diseases through multimodal mechanisms, including antioxidant, anti-inflammatory, anti-apoptotic, and neuroprotective actions.

**Figure 6:**
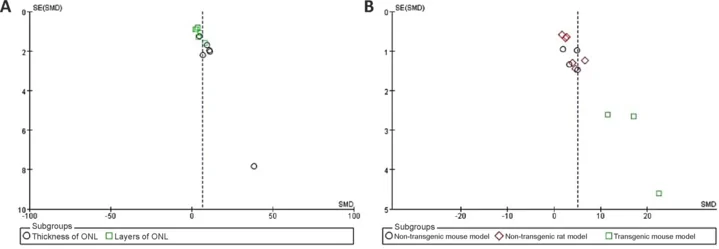
Funnel plots for publication bias assessment. (A) Funnel plot for outer nuclear layer (ONL) thickness, showing the distribution of effect sizes (standardized mean differences, SMD) against their standard errors. (B) Funnel plot for b-wave amplitude, illustrating the relationship between effect sizes and their standard errors. Both plots include dots representing the primary studies included in the meta-analysis.

#### Antioxidant effect

Six studies have documented the capacity of LBP to mitigate oxidative stress in models of optic nerve injury. Following LBP intervention, a reduction in ROS was observed (He et al., 2014). Concurrently, an increase in antioxidant enzymes, including SOD, GSH-Px, and CAT, was reported (Li et al., 2013; Wang et al., 2017; Au et al., 2022), along with an increase in the expression of Nrf2/HO-1 (Pan et al., 2019).

#### Anti-inflammatory effect

LBP has been demonstrated to exert significant anti-inflammatory effects, with the capacity to inhibit the occurrence and development of inflammatory responses. This study presents five experimental results showing that LBP can inhibit and reduce the inflammatory response in optic nerve diseases. Two studies demonstrated that LBP can inhibit the release of pro-inflammatory factors (Wang et al., 2014; Kong et al., 2024), including TNF-α, IL-1β, and IL-6. Two additional studies corroborated the suppressive effect of LBP treatment on these inflammatory factors (Li et al., 2015; Mi et al., 2020). Furthermore, two studies reported a decrease in Iba-1, a marker of microglial activation (Wang et al., 2014; Li et al., 2019), while other studies also noted a reduction in CD68, a marker associated with macrophage activity (Wang et al., 2014).

#### Antiapoptotic anti-apoptosis

This study presents five experimental results demonstrating that LBP reduces apoptosis by increasing the expression of apoptotic proteins Bcl-2 and decreasing the expression of apoptotic proteins Bax and Caspase.

#### Neuroprotection

Several experimental studies examined the impact of LBP on glial cells in the retina. Among them, seven studies indicated a significant decrease in GFAP levels in the LBP group, suggesting inhibition of astrocyte activation (Yao et al., 2018; Pan et al., 2019). Additionally, previous studies showed that Iba-1 levels were also significantly reduced (Li et al., 2015; Mi et al., 2020), indicating inhibition of microglial activation following LBP treatment. Furthermore, a reduction in glutamine synthetase was observed, suggesting that LBP may have the ability to regulate glial cell activity and lessen neuroinflammation in the retina.

### Publication bias analysis

A formal quantitative assessment of publication bias (e.g., funnel plot asymmetry or Egger’s test) was not conducted for the meta-analyses of ONL thickness (*n* = 5 studies) and *b*-wave amplitude (*n* = 6 studies) due to the limited number of included studies (fewer than 10). This threshold is widely recognized as the minimum required for reliable statistical testing of publication bias. However, funnel plots were generated to provide a visual assessment of potential publication bias (**Figure 5**). While the funnel plot did not reveal any overt indications of bias, the limited number of studies included in the analysis precludes definitive conclusions. A qualitative assessment revealed no overt indications of bias: (1) Effect sizes for both outcomes demonstrated consistent directional trends across all studies; (2) No studies reported extreme effect magnitudes disproportionate to their sample sizes; and (3) The included publications represented diverse research groups and geographic regions. Nonetheless, the possibility of unpublished negative results cannot be excluded, particularly given the observed heterogeneity (*I*^2^ > 80%), which may reflect selective reporting of positive outcomes within the current literature.

## Discussion

### Evidence summary

This systematic review retrieved 27 studies through literature searches, and the meta-analysis conducted in this study provides robust quantitative evidence supporting the retinoprotective efficacy of LBP. The meta-analysis showed that LBP significantly increased ONL thickness and b-wave amplitude, with consistent positive effects on both measures across diverse rodent models, underscoring the reliability of LBP’s therapeutic potential. Notably, the effect sizes for ONL thickness improvement were substantial, indicating a strong protective effect on photoreceptor survival. Additionally, the restoration of b-wave amplitude suggests that LBP not only preserves retinal structure but also functionally rescues inner retinal circuitry. This dual benefit is particularly significant given that many existing therapies focus solely on structural or functional aspects, whereas LBP appears to offer comprehensive protection. The low probability of publication bias, as qualitatively assessed, further strengthens the validity of these findings. These results indicate that LBP has a protective effect against optic nerve damage in various retinal disease models, with several mechanisms, including antioxidant, anti-inflammatory, and anti-apoptotic effects, responsible for its retinal protective activity. However, several limitations of current research on LBP have been identified. For example, methodological limitations include inadequate allocation concealment and a lack of blinding for caregivers, investigators, and outcome assessors, resulting in a risk of performance and detection bias (SYRCLE Domains 3, 5, 6). Additionally, the high heterogeneity observed in both outcomes highlights the need for standardized methodologies in future studies to reduce variability and enhance comparability.

#### Application of rodent models of retinal diseases

Six retinal disease models, including glaucoma, DR, I/R, AMD, RP, and TON, have been used to evaluate LBP activity. The animal models of glaucoma included in this study were primarily SD rats (76.92%) and C57BL/6N mice (23.08%), with no observed interventional effect of LBP in DBA/2J transgenic mice, a model of spontaneous glaucoma that mimics human angle-closure glaucoma. The DBA/2J transgenic mouse model is driven by specific gene mutations, and chronic intraocular pressure elevation leads to progressive retinal ganglion cell loss and optic nerve damage, closely resembling human primary glaucoma (Saleh et al., 2007; Bierlein et al., 2022). Thus, the DBA/2J transgenic mouse model could minimize key experimental biases and enhance clinical relevance. Its slow, age-dependent disease progression provides a reliable platform for assessing long-term protective efficacy under real-world chronic conditions, effectively preventing the temporal bias seen in rapid injury models, where short-term effects might be overestimated and long-term therapeutic value underestimated. Therefore, it is recommended that the role of LBP in the DBA/2J transgenic mouse model be further explored. The rat retina is large, facilitating fundus imaging and pathological section analysis; consequently, all diabetic retinopathy animal models were SD rats, induced with STZ to mimic chronic hyperglycemia, leading to retinal microvascular leakage and neurodegenerative lesions. Animal models of retinitis pigmentosa include genetic models, such as rd1 and rd10 mice, and non-genetic models, such as MNU-induced SD rats. The rd1 and rd10 mice exhibit a well-defined genotype-phenotype correlation and high reproducibility, while the MNU model demonstrates an adjustable pathological process with a wide range of applicability, providing an ideal platform for investigating non-genetic mechanisms and intervention strategies (Sarunic et al., 2010; Fradot et al., 2024; Grannonico et al., 2024).

#### Evaluation value of outer nuclear layer thickness in Lycium barbarum polysaccharides treatment of retinal diseases

The ONL, densely packed with photoreceptor somata, is indispensable for efficient visual signal transmission (Lee et al., 2023). In blinding disorders such as glaucoma, diabetic retinopathy, and retinitis pigmentosa, progressive ONL thinning is the cardinal morphologic correlate of photoreceptor apoptosis (Sayo et al., 2018; Otsuka et al., 2022). This irreversible deterioration strongly predicts visual acuity loss and visual field defects, rendering ONL thickness a gold-standard metric for assessing neuroprotective efficacy. Furthermore, the ONL exhibits high sensitivity and specificity across rodent models of phototoxicity, genetic mutation, and ischemia-reperfusion injury, thereby facilitating the cross-model integration of drug effects. Accordingly, we selected ONL thickness as the objective outcome measure to quantify the protective potency of LBPs on photoreceptors in this meta-analysis. Our findings demonstrate that LBP treatment significantly increases ONL thickness. Nevertheless, subgroup analyses revealed higher heterogeneity for thickness-based quantification (*I*^2^ = 82%) than for cell-layer counts (*I*^2^ = 60%). Apart from extreme statistical outliers in individual studies, this discrepancy likely reflects the greater stability and lower measurement error inherent in cell-layer counting. Across rodent species, the number of ONL cell layers remains relatively constant, whereas soma size exhibits appreciable variability. Therefore, we strongly recommend that future investigations employ both metrics concurrently to maximize inter-study comparability and minimize analytical bias.

#### Evaluation value of b-wave in Lycium barbarum polysaccharides treatment of retinal diseases

The b-wave amplitude of the electroretinogram is the principal electrophysiological readout for assessing inner retinal circuit integrity (Brabec et al., 2023; Chan et al., 2024). Its attenuation precedes morphological damage and thus serves as an early sentinel of disease progression. Generated by trans-synaptic signaling between bipolar and Müller cells, a reduced b-wave amplitude directly signifies bipolar cell dysfunction or glial homeostatic failure (Dmitriev et al., 2021). In diabetic retinopathy, the extent of b-wave decline correlates tightly with microvascular lesions (Sugasini et al., 2024); in glaucoma models, b-wave abnormalities emerge before retinal ganglion cell loss, providing a sensitive predictor of impending visual field defects (Ye et al., 2025). Critically, b-wave generation is highly conserved across species, making it a translational bridge between preclinical research and clinical application. Our meta-analysis demonstrates that LBP treatment effectively rescues disease-associated reductions in b-wave amplitude. As a relative metric, the b-wave is susceptible to both systematic and random errors introduced at multiple measurement stages. For instance, variations in stimulus luminance produce amplitude differences that persist even when identical strains are assessed in different laboratories. Animal age is another source of heterogeneity identified in this review. Notably, rd10 transgenic mice, whose disease onset is early, are typically tested at 1 month of age. This developmental stage renders the b-wave more labile, explaining the greater variability observed in this subgroup’s amplitude data.

#### Mechanism of Lycium barbarum polysaccharides in treating retinal diseases

LBPs have been shown to possess antioxidant activity by eliminating free radicals, stimulating antioxidant enzyme activities (such as SOD, GPx, and CAT), increasing glutathione synthesis, activating mitochondria, and improving cell membrane stability. LBPs can also inhibit the secretion of pro-inflammatory factors such as TNF-α, IL-1β, and IL-6, as well as other inflammatory mediators. Studies have demonstrated that nitric oxide and cyclooxygenase-2 can block the NF-κB and STAT3 pathways, thereby regulating immune cell activity and the inflammatory response (Wang et al., 2014; Kong et al., 2024). Furthermore, LBPs can alleviate optic nerve cell apoptosis by regulating apoptotic proteins (Liu et al., 2020; Ni et al., 2024). However, different retinal disease models may exhibit varying responses to the retinal neuroprotective mechanisms of LBPs. The multitarget mechanisms of LBP in retinal disease therapy demonstrate pronounced model-dependent interactions and variations. Its antioxidant, anti-inflammatory, and anti-apoptotic pathways do not act in isolation but form a synergistic network, with the dominant mechanism differing across pathological contexts. For example, in I/R and glaucoma models, LBPs enhance endogenous antioxidant enzymes such as SOD and GPx via Nrf2/HO-1 activation while simultaneously suppressing mitochondrial ROS generation. This antioxidant effect directly mitigates oxidative stress-induced apoptosis and indirectly inhibits NF-κB-driven inflammatory cascades. In contrast, anti-apoptotic effects are more prominent in hereditary retinal degeneration and light-damage models. Notably, in diabetic retinopathy models, LBPs stabilize the blood-retinal barrier through the Rho/ROCK pathway, with their anti-inflammatory and antioxidant mechanisms jointly reducing vascular leakage and gliosis. Therefore, further studies are needed to elucidate the specific molecular mechanisms and the regulation of related signaling pathways in order to identify more precise targets and pathways in the field of retinal neuroprotection. Considering the complexity of animal models in dissecting molecular mechanisms, new technologies such as human retinal organoids and single-cell transcriptomic analysis can be exploited to some extent in retinal disease studies and the investigation of pharmacological mechanisms of action. The dosage and duration of LBP treatment in different animal models and retinal disease models have been inconsistent. Thus, future work must clarify the efficacy and safety of treatment dosages and timing, which are crucial for the wider clinical application of LBPs and the development of new products.

Despite the compelling evidence for multi-target mechanisms of LBP, the current body of research exhibits significant gaps in mechanistic understanding. Most studies have focused on a limited set of pathways, such as antioxidant and anti-inflammatory effects, but have not fully explored the interplay between these pathways or their relative contributions in different disease contexts. For instance, while Nrf2/HO-1 activation is frequently reported, the upstream regulators and downstream effectors of this pathway in the retina remain poorly characterized. Additionally, the role of LBP in modulating glial-neuronal interactions, which are critical for retinal homeostasis, warrants deeper investigation. The lack of temporal data on mechanism activation also limits insights into the dynamics of the action of LBP. Future studies should employ time-course experiments to delineate the sequence of molecular events following LBP administration. Moreover, integrating omics technologies, such as transcriptomics and proteomics, could uncover novel targets and provide a systems-level understanding of the mechanisms of LBP. This approach would not only validate existing findings but also identify new avenues for therapeutic development.

The analysis of current studies reveals that the majority of LBP administration in animals is conducted via gavage. Considering the low bioavailability of polysaccharides, new pharmaceutical preparations of LBP, such as ophthalmic drops and ointments, should be considered in future studies. Meanwhile, advanced drug delivery technologies, including nanoparticle preparations, can be employed for retinal-targeted delivery to improve therapeutic efficacy.

### Limitations

Several limitations of this systematic review and meta-analysis merit attention. Among the 27 included studies, 92.6% failed to describe allocation concealment, and 85.2% omitted assessor blinding, introducing risks of performance and detection bias that may inflate treatment-effect estimates. Encouragingly, recently published reports demonstrate a discernible trend toward greater methodological rigor, with more comprehensive baseline characteristic reporting. The scope and depth of outcome evaluation have also expanded over time—from rudimentary structural indices to comprehensive functional measures and increasingly detailed mechanistic explorations.

A notable concern is the concentration of investigations within a limited number of research groups; approximately half (13/27) of the studies originated from a single laboratory network. Such clustering may embed protocol-standardization biases or methodological idiosyncrasies that restrict the generalizability of pooled estimates. At the review level, our search was confined to PubMed and Web of Science, capturing predominantly English- and Chinese-language publications. This strategy may have overlooked relevant preclinical studies reported in other languages (e.g., Japanese, Korean, or German) from regions with active neuroprotective-agent research programs. While linguistic bias is an acknowledged constraint, our stringent inclusion/exclusion criteria—especially the requirements for full-text availability, controlled study design, and clearly reported outcomes—were essential to safeguard analytical robustness, even if they resulted in the exclusion of potentially relevant but methodologically weaker reports.

Another major limitation is the heterogeneity in LBP preparation and characterization across studies. LBP is a complex mixture of polysaccharides with varying molecular weights and compositions, which can influence its bioactivity. However, few studies provided detailed information on the chemical properties of the LBP used, such as monosaccharide composition, glycosidic linkages, or molecular weight distribution. This lack of standardization complicates comparisons between studies and may contribute to the observed variability in efficacy. Furthermore, the doses administered ranged widely from 1 to 400 mg/kg, without a clear rationale for dose selection, and treatment durations varied from 1 to 20 weeks. This inconsistency precludes the establishment of a dose-response relationship and optimal treatment regimen. Additionally, the predominance of acute models over chronic models may overestimate the efficacy of LBP in long-term degenerative conditions. For example, most glaucoma models used acute I/R or hypertension induction, which do not fully recapitulate the slow progression of human glaucoma. The underrepresentation of genetic models, such as DBA/2J mice, further limits the clinical relevance of the findings. These limitations emphasize the need for more rigorous study designs that include standardized LBP characterization, dose-ranging studies, and clinically relevant animal models.

As the field advances and the therapeutic relevance of LBP in retinal disorders is increasingly recognized, we anticipate a growing body of high-quality literature that will enable more comprehensive and objective analyses.

### Applicability and implications for future research

The integrated preclinical evidence from this study positions LBP as a highly promising candidate drug with significant translational potential in the field of retinal neuroprotection. Its multi-target synergistic mechanisms—simultaneously targeting oxidative stress, inflammation, apoptosis, and glial dysregulation—provide strategic advantages over single-pathway therapies, particularly for complex, multifactorial retinal diseases such as diabetic retinopathy and glaucoma. Given the established safety profile of wolfberry as a traditional dietary ingredient, LBP possesses inherent advantages for clinical development. Looking ahead, research improvements should focus on four key areas. First, methodologically high standards for randomization, blinding, and allocation concealment should be adopted, along with the introduction of animal models that better simulate the chronicity and complexity of human diseases (e.g., the DBA/2J mouse glaucoma model) to strengthen the evidence base. Second, dose-effect optimization is needed. Given the wide dose range (1–400 mg/kg) and inconsistent treatment durations (1–20 weeks) in existing studies, systematic dose-response studies are urgently required. Physiologically based pharmacokinetic (PBPK) modeling can further predict dose-effect relationships to provide quantitative evidence for clinical dose selection. Third, innovations in delivery technology are essential, including the development of nanocarriers, intravitreal sustained-release formulations, or topical eye drops to overcome the oral bioavailability limitations of polysaccharides and achieve precise retinal delivery.

### Conclusion

LBP demonstrates strong potential as a new therapeutic option for the treatment of retinal diseases, according to findings from extensive animal studies. However, limitations in the design of these animal experiments raise caution regarding the further development of LBP. More carefully designed animal studies with focused disease models would provide robust evidence to justify the initiation of clinical trials in humans.

## Data Availability

*No additional data are available.*

## References

[R1] Agarwal R, Agarwal P (2017). Rodent models of glaucoma and their applicability for drug discovery. Expert Opin Drug Discov.

[R2] Ali Z, Ayub A, Lin Y, Anis S, Khan I, Younas S, Tahir RA, Wang S, Li J (2025). Lycium b arbarum’s diabetes secrets: a comprehensive review of cellular, molecular, and epigenetic targets with immune modulation and microbiome influence. J Pharm Anal.

[R3] Al-Wraikat M, Zhang L, Li L, Abubaker MA, Liu Y (2024). Recent advances in wolfberry polysaccharides and whey protein-based biopolymers for regulating the diversity of gut microbiota and its mechanism: A review. Int J Biol Macromol.

[R4] Ambati J, Anand A, Sakurai E, Fernandez S, Rollins BJ, Kuziel WA, Lynn BC, Ambati BK (2004). An animal model of atrophic and neovascular age–related macular degeneration in Ccl2–/– or Ccr2–/– mice. Invest Ophthalmol Vis Sci.

[R5] Antonetti DA, Lin C-M, Shanmugam S, Hager H, Cao M, Liu X, Dreffs A, Habash A, Abcouwer SF (2024). Diabetes renders photoreceptors susceptible to retinal ischemia-reperfusion injury. Invest Ophthalmol Vis Sci.

[R6] Au NPB, Kumar G, Asthana P, Gao F, Kawaguchi R, Chang RCC, So KF, Hu Y, Geschwind DH, Coppola G, Ma CHE (2022). Clinically relevant small-molecule promotes nerve repair and visual function recovery. NPJ Regen Med.

[R7] Bierlein ER, Smith JC, Van Hook MJ (2022). Mechanism for altered dark-adapted electroretinogram responses in DBA/2J mice includes pupil dilation deficits. Curr Eye Res.

[R8] Brabec M, Constable PA, Thompson DA, Marmolejo-Ramos F (2023). Group comparisons of the individual electroretinogram time trajectories for the ascending limb of the b-wave using a raw and registered time series. BMC Res Notes.

[R9] Burdová MČ, Štěpánková J, Pourová RK, Mahelková G, Hložánek M, Kožner P, Dotřelová D (2023). Long-term anatomical and functional outcomes of surgical treatment of retinal complications in children and adolescents with Stickler syndrome between 2004 and 2021. Graefes Arch Clin Exp Ophthalmol Albrecht Von Graefes Arch Klin Exp Ophthalmol.

[R10] Cammalleri M, Bagnoli P (2025). The long-standing problem of proliferative retinopathies: current understanding and critical cues. Cells.

[R11] Chan HC, Chang RCC, Koon-Ching Ip A, Chiu K, Yuen WH, Zee SY, So KF (2007). Neuroprotective effects of Lycium barbarum Lynn on protecting retinal ganglion cells in an ocular hypertension model of glaucoma. Exp Neurol.

[R12] Chan SSH, Choi KY, Chan HHL (2024). Paediatric norms for photopic electroretinogram testing based on a large cohort of Chinese preschool children. BMJ Open Ophthalmol.

[R13] Chen Y, Tang S, Huang Y, Deng J, Chen X, Qi Y, Xiao H, Li Y, Li H, Guan H (2025). Global burden of blindness or visually impairment attributable to diabetic retinopathy in the adults aged 70 years and older, 1990–2021: results from the global burden of disease study in 2021. Diabetes Res Clin Pract.

[R14] Chen YY, Liu QP, An P, Jia M, Luan X, Tang JY, Zhang H (2022). Ginsenoside Rd: a promising natural neuroprotective agent. Phytomed Int J Phytother Phytopharm.

[R15] Cheng C, Nguyen MN, Nayernama A, Jones SC, Brave M, Agrawal S, Amiri-Kordestani L, Woronow D (2021). Arterial aneurysm and dissection with systemic vascular endothelial growth factor inhibitors: a review of cases reported to the FDA Adverse Event Reporting System and published in the literature. Vasc Med Lond Engl.

[R16] Chiu K, Chan HC, Yeung SC, Yuen WH, Zee SY, Chang RCC, So KF (2009). Modulation of microglia by Wolfberry on the survival of retinal ganglion cells in a rat ocular hypertension model. J Ocul Biol Dis Infor.

[R17] Chiu K, Zhou Y, Yeung SC, Lok CKM, Chan OOC, Chang RCC, So KF, Chiu JF (2010). Up-regulation of crystallins is involved in the neuroprotective effect of wolfberry on survival of retinal ganglion cells in rat ocular hypertension model. J Cell Biochem.

[R18] Chong RS, Busoy JMF, Tan B, Yeo SW, Lee YS, Barathi AV, Crowston JG, Schmetterer L (2020). A minimally invasive experimental model of acute ocular hypertension with acute angle closure characteristics. Transl Vis Sci Technol.

[R19] Chronopoulos P, Manicam C, Zadeh JK, Laspas P, Unkrig JC, Göbel ML, Musayeva A, Pfeiffer N, Oelze M, Daiber A, Li H, Xia N, Gericke A (2023). Effects of resveratrol on vascular function in retinal ischemia-reperfusion injury. Antioxid Basel Switz.

[R20] Chu PHW, Li HY, Chin MP, So K, Chan HHL (2013). Effect of lyciumbarbarum (wolfberry) polysaccharides on preserving retinal function after partial optic nerve transection. PLoS One.

[R21] Distefano LN, Garcia-Arumi J, Martinez-Castillo V, Boixadera A (2017). Combination of anti-VEGF and laser photocoagulation for diabetic macular edema: a review. J Ophthalmol.

[R22] Dmitriev AV, Dmitriev AA, Linsenmeier RA (2021). K+-dependent Müller cell-generated components of the electroretinogram. Vis Neurosci.

[R23] Fradot V, Augustin S, Fontaine V, Marazova K, Guillonneau X, Sahel JA, Picaud S (2024). Rodent models of retinal degeneration: from purified cells in culture to living animals. Cold Spring Harb Perspect Med.

[R24] Grannonico M, Miller DA, Liu M, Krause MA, Savier E, Erisir A, Netland PA, Cang J, Zhang HF, Liu X (2024). Comparative in vivo imaging of retinal structures in tree shrews, humans, and mice. eNeuro.

[R25] He M, Pan H, Chang RCC, So KF, Brecha NC, Pu M (2014). Activation of the Nrf2/HO-1 antioxidant pathway contributes to the protective effects of Lycium barbarum polysaccharides in the rodent retina after ischemia-reperfusion-induced damage. PLoS One.

[R26] Hooijmans CR, Rovers MM, de Vries RBM, Leenaars M, Ritskes-Hoitinga M, Langendam MW (2014). SYRCLE’s risk of bias tool for animal studies. BMC Med Res Methodol.

[R27] Hu H, Wu L, Hu X, Wang M, Sun Y, Zhang G, Wang Y, Yi P, Zhang Z (2025). Neuroprotective and intraocular pressure lowering effects of dual-functional memantine nitrate MN-08 on the experimental models of glaucoma. Sci Rep.

[R28] Hu X, Mu L, Zhu L, Chang X, Nie L, Wang L, Li G (2021). Lycium barbarum polysaccharides attenuate cardiovascular oxidative stress injury by enhancing the Keap1/Nrf2 signaling pathway in exhaustive exercise rats. Mol Med Rep.

[R29] Joshi P, Dangwal A, Guleria I, Kothari S, Singh P, Kalra JM, Jakhmola V (2022). Glaucoma in adults-diagnosis, management, and prediagnosis to end-stage, categorizing glaucoma’s stages: a review. J Curr Glaucoma Pract.

[R30] Kang JM, Tanna AP (2021). Glaucoma. Med Clin North Am.

[R31] Khan I, Ramzan F, Tayyab H, Damji KF (2025). Rekindling vision: innovative strategies for treating retinal degeneration. Int J Mol Sci.

[R32] Kong Q, Han X, Cheng H, Liu J, Zhang H, Dong T, Chen J, So KF, Mi X, Xu Y, Tang S (2024). Lycium barbarum glycopeptide (wolfberry extract) slows N-methyl-N-nitrosourea-induced degradation of photoreceptors. Neural Regen Res.

[R33] Kovács-Valasek A, Rák T, Pöstyéni E, Csutak A, Gábriel R (2023). Three major causes of metabolic retinal degenerations and three ways to avoid them. Int J Mol Sci.

[R34] Lakshmanan Y, Wong FSY, Yu WY, Li SZC, Choi KY, So KF, Chan HHL (2019). Lycium Barbarumpolysaccharides rescue neurodegeneration in an acute ocular hypertension rat model under pre- and posttreatment conditions. Invest Ophthalmol Vis Sci.

[R35] Lakshmanan Y, Wong FSY, Zuo B, So KF, Bui BV, Chan HHL (2019). Posttreatment Intervention WithLycium Barbarumpolysaccharides is neuroprotective in a rat model of chronic ocular hypertension. InvestigOpthalmology Vis Sci.

[R36] Lambert V, Lecomte J, Hansen S, Blacher S, Gonzalez M-LA, Struman I, Sounni NE, Rozet E, de Tullio P, Foidart JM, Rakic J-M, Noel A (2013). Laser-induced choroidal neovascularization model to study age-related macular degeneration in mice. Nat Protoc.

[R37] Latifi-Navid H, Barzegar Behrooz A, Jamehdor S, Davari M, Latifinavid M, Zolfaghari N, Piroozmand S, Taghizadeh S, Bourbour M, Shemshaki G, Latifi-Navid S, Arab SS, Soheili Z-S, Ahmadieh H, Sheibani N (2023). Construction of an exudative age-related macular degeneration diagnostic and therapeutic molecular network using multi-layer network analysis, a fuzzy logic model, and deep learning techniques: are retinal and brain neurodegenerative disorders related?. Pharm Basel Switz.

[R38] Lee H, Jang H, Chae JB, Lee HK, Son C, Park CW, Ha T, Yum M, Park S, Hong SW, Lee SY, Lyu J, Lee S, Lee DK, Chung H (2025). In vivo efficacy of NRL knockdown with cell-penetrating siRNA in retinal degeneration. Sci Rep.

[R39] Lee S, Kim KT, Kim DY, Chae JB, Seo EJ (2023). Outer nuclear layer recovery as a predictor of visual prognosis in type 1 choroidal neovascularization of neovascular age-related macular degeneration. Sci Rep.

[R40] Li H, Liang Y, Chiu K, Yuan Q, Lin B, Chang RCC, So KF (2013). Lycium barbarum (wolfberry) reduces secondary degeneration and oxidative stress, and inhibits JNK pathway in retina after partial optic nerve. PLoS One.

[R41] Li HY, Ruan YW, Kau PWF, Chiu K, Chang RCC, Chan HHL, So KF (2015). Effect of Lycium barbarum (Wolfberry) on alleviating axonal degeneration after partial optic nerve transection. Cell Transplant.

[R42] Li HY, Huang M, Luo QY, Hong X, Ramakrishna S, So KF (2019). Lycium barbarum (wolfberry) increases retinal ganglion cell survival and affects both microglia/macrophage polarization and autophagy after rat partial optic nerve transection. Cell Transplant.

[R43] Li J, Yu Y, Zhang Y, Zhou Y, Ding S, Dong S, Jin S, Li Q (2024). Flavonoids derived from Chinese medicine: potential neuroprotective agents. Am J Chin Med.

[R44] Li SY, Yang D, Yeung CM, Yu WY, Chang RCC, So KF, Wong D, Lo ACY (2011). Lycium barbarum polysaccharides reduce neuronal damage, blood-retinal barrier disruption and oxidative stress in retinal ischemia/reperfusion injury. PLoS One.

[R45] Liang R, Zhao Q, Zhu Q, He X, Gao M, Wang Y (2021). Lycium barbarum polysaccharide protects ARPE-19 cells against H2O2-induced oxidative stress via the Nrf2/HO-1 pathway. Mol Med Rep.

[R46] Liu F, Zhang J, Xiang Z, Xu D, So KF, Vardi N, Xu Y (2018). Lycium barbarum polysaccharides protect retina in rd1 mice during photoreceptor degeneration. Invest Ophthalmol Vis Sci.

[R47] Liu L, Sha XY, Wu YN, Chen MT, Zhong JX (2020). Lycium barbarum polysaccharides protects retinal ganglion cells against oxidative stress injury. Neural Regen Res.

[R48] Masini S, Bruschi M, Menotta M, Canonico B, Montanari M, Ligi D, Monittola F, Mannello F, Piersanti G, Crinelli R, Magnani M, Fraternale A (2025). Redox modulation by a synthetic thiol compound reduces LPS-induced pro-inflammatory cytokine expression in macrophages via AP-1/NLRP3 axis and influences the crosstalk with endothelial cells. Free Radic Res.

[R49] Mehta NJ, Mehta SN (2024). Nanotechnology in retinal disease: current concepts and future directions. J Assoc Ocul Pharmacol Ther.

[R50] Mi X, Zhong J, Chang RCC, So KF (2013). Research advances on the usage of traditional Chinese medicine for neuroprotection in glaucoma. J Integr Med.

[R51] Mi XS, Chiu K, Van G, Leung JWC, Lo ACY, Chung SK, Chang RCC, So KF (2012). Effect of Lycium barbarum Polysaccharides on the expression of endothelin-1 and its receptors in an ocular hypertension model of rat glaucoma. Neural Regen Res.

[R52] Mi XS, Feng Q, Lo ACY, Chang RCC, Lin B, Chung SK, So KF (2012). Protection of retinal ganglion cells and retinal vasculature by lyciumbarbarum polysaccharides in a mouse model of acute ocular hypertension. PLoS One.

[R53] Mi XS, Feng Q, Lo ACY, Chang RCC, Chung SK, So KF (2020). Lycium barbarum polysaccharides related RAGE and Aβ levels in the retina of mice with acute ocular hypertension and promote maintenance of blood retinal barrier. Neural Regen Res.

[R54] Ni J, Au M, Kong H, Wang X, Wen C (2021). Lycium barbarum polysaccharides in ageing and its potential use for prevention and treatment of osteoarthritis: a systematic review. BMC Complement Med Ther.

[R55] Ni Y, Hu Y, Zhu L, Jiang X, Zhang H, Liu J, Zhao Y (2024). Lycium barbarum polysaccharide-derived nanoparticles protect visual function by inhibiting RGC ferroptosis and microglial activation in retinal ischemia‒reperfusion mice. Adv Healthc Mater.

[R56] Oosthuyse B (2001). Deletion of the hypoxia-response element in the vascular endothelial growth factor promoter causes motor neuron degeneration. Nat Genet.

[R57] Oshitari T (2024). Translational research and therapies for neuroprotection and regeneration of the optic nerve and retina: a narrative review. Int J Mol Sci.

[R58] Otsuka Y, Oishi A, Miyata M, Uji A, Oishi M, Hasegawa T, Numa S, Ikeda HO, Tsujikawa A (2022). Evaluation of outer nuclear layer overshadowed by retinal vessels in retinitis pigmentosa. Eye Lond Engl.

[R59] Page MJ (2021). PRISMA 2020 explanation and elaboration: updated guidance and exemplars for reporting systematic reviews. BMJ.

[R60] Pan H, Shi Z, Yang TG, Yu LM, Xu AL (2019). The protective effects of lyciumbarbarum polysaccharides on retinal neurons in diabetic rats and its mechanism. Zhongguo Ying Yong Sheng Li Xue Za Zhi.

[R61] Parvez MK (2018). Natural or plant products for the treatment of neurological disorders: current knowledge. Curr Drug Metab.

[R62] Patel C, Pande S, Sagathia V, Ranch K, Beladiya J, Boddu SHS, Jacob S, Al-Tabakha MM, Hassan N, Shahwan M (2023). Nanocarriers for the delivery of neuroprotective agents in the treatment of ocular neurodegenerative diseases. Pharmaceutics.

[R63] Petersen-Jones SM, Komáromy AM (2024). Canine and feline models of inherited retinal diseases. Cold Spring Harb Perspect Med.

[R64] Qi B, Ji Q, Wen Y, Liu L, Guo X, Hou G, Wang G, Zhong J (2014). Lycium barbarum polysaccharides protect human lens epithelial cells against oxidative stress-induced apoptosis and senescence. PLoS One.

[R65] Qi Y, Duan G, Fan G, Peng N (2022). Effect of Lycium barbarum polysaccharides on cell signal transduction pathways. Biomed Pharmacother.

[R66] Rong Y, Li J, He J, Zhang D, Wu J, Liu H, Li T, Xu P, Chang Q, Wu J (2025). Genotype-specific retinal and choroidal perfusion patterns in inherited retinal diseases: an SS-OCTA analysis. Int J Retina Vitr.

[R67] Sabanayagam C, Yip W, Ting DSW, Tan G, Wong TY (2016). Ten emerging trends in the epidemiology of diabetic retinopathy. Ophthalmic Epidemiol.

[R68] Saleh M, Nagaraju M, Porciatti V (2007). Longitudinal evaluation of retinal ganglion cell function and IOP in the DBA/2J mouse model of glaucoma. Invest Ophthalmol Vis Sci.

[R69] Sarunic MV, Yazdanpanah A, Gibson E, Xu J, Bai Y, Lee S, Saragovi HU, Beg MF (2010). Longitudinal study of retinal degeneration in a rat using spectral domain optical coherence tomography. Opt Express.

[R70] Sato T, Ishikawa M, Izumi Y, Shibata N, Sato K, Ohno-Oishi M, Tawarayama H, Kunikata H, Zorumski CF, Nakazawa T (2025). Involvement of microglia in retinal ganglion cell injury induced by IOP elevation in a rat ex vivo acute glaucoma model. Biomedicines.

[R71] Sayo A, Ueno S, Kominami T, Okado S, Inooka D, Komori S, Terasaki H (2018). Significant relationship of visual field sensitivity in central 10° to thickness of retinal layers in retinitis pigmentosa. Invest Ophthalmol Vis Sci.

[R72] Schmid H, Renner M, Dick HB, Joachim SC (2014). Loss of inner retinal neurons after retinal ischemia in rats. Invest Ophthalmol Vis Sci.

[R73] Shastry BS (2008). Evaluation of the common variants of the ABCA4 gene in families with Stargardt disease and autosomal recessive retinitis pigmentosa. Int J Mol Med.

[R74] Simmonds M, Walton M, Hodgson R, Llewellyn A, Walker R, Fulbright H, Bojke L, Stewart L, Dias S, Rush T, Lawrenson J, Peto T, Steel D (2025). Anti-VEGF drugs compared with laser photocoagulation for the treatment of diabetic retinopathy: a systematic review and economic analysis. Health Technol Assess Winch Engl.

[R75] Stitt AW, Curtis TM, Chen M, Medina RJ, McKay GJ, Jenkins A, Gardiner TA, Lyons TJ, Hammes H-P, Simó R, Lois N (2016). The progress in understanding and treatment of diabetic retinopathy. Prog Retin Eye Res.

[R76] Sugasini D, Yalagala PCR, Park JC, Ma G, Farooq Z, Baccouche B, Sawant OB, McAnany JJ, Yao X, Kazlauskas A, Layden BT, Subbaiah PV (2024). Retinal docosahexaenoic acid is significantly reduced in diabetic humans and mice: possible relationship to diabetic retinopathy. Invest Ophthalmol Vis Sci.

[R77] Tang L, Bao S, Du Y, Jiang Z, Wuliji AO, Ren X, Zhang C, Chu H, Kong L, Ma H (2018). Antioxidant effects of Lycium barbarum polysaccharides on photoreceptor degeneration in the light-exposed mouse retina. Biomed Pharmacother Biomedecine Pharmacother.

[R78] Tang L, Xu GT, Zhang JF (2023). Inflammation in diabetic retinopathy: possible roles in pathogenesis and potential implications for therapy. Neural Regen Res.

[R79] Tham YC, Li X, Wong TY, Quigley HA, Aung T, Cheng C-Y (2014). Global prevalence of glaucoma and projections of glaucoma burden through 2040: a systematic review and meta-analysis. Ophthalmology.

[R80] Tsai JC (2020). Innovative IOP-independent neuroprotection and neuroregeneration strategies in the pipeline for glaucoma. J Ophthalmol.

[R81] Wang J, Yao Y, Liu X, Wang K, Zhou Q, Tang Y (2019). Protective effects of lyciumbarbarum polysaccharides on blood-retinal barrier via ROCK1 pathway in diabetic rats. Am J Transl Res.

[R82] Wang K, Xiao J, Peng B, Xing F, So KF, Tipoe GL, Lin B (2014). Retinal structure and function preservation by polysaccharides of wolfberry in a mouse model of retinal degeneration. Sci Rep.

[R83] Wang L, Chen Y, Liu Y, Wei J, Miao L, Chang Z, Jia M, Liu L, Liang X, Zhang Y (2025). Traditional chinese medicine for cerebral ischemia-reperfusion injury prevention: molecular mechanisms and future perspectives. Am J Chin Med.

[R84] Wang S, Xu Z, Zhao Y, Liu C (2020). On the protective effect of lyciumbarbarum polysaccharide (LBP) on optic nerve tissue and retinal ganglion cells. J Biomater Tissue Eng.

[R85] Wang X, Li M, Diao K, Wang Y, Chen H, Zhao Z, Li Y, Jia X, Wang H, Zheng F, Xia Z, Han L, Zhang M (2023). Deferoxamine attenuates visual impairment in retinal ischemia‒reperfusion via inhibiting ferroptosis. Sci Rep.

[R86] Wang Y, Ding L, Li Y, Guan C, Guo J (2017). Lycium barbarum polysaccharides can reduce the oxidative damage of the retinal nerve cells in diabetic rats. Int J Clin Exp Med.

[R87] Watson N, Al-Samkari H (2021). Thrombotic and bleeding risk of angiogenesis inhibitors in patients with and without malignancy. J Thromb Haemost.

[R88] Wei M, Zhang G, Huang Z, Ding X, Sun Q, Zhang Y, Zhu R, Guan H, Ji M (2023). ATP-P2X7R-mediated microglia senescence aggravates retinal ganglion cell injury in chronic ocular hypertension. J Neuroinflammation.

[R89] Wei S, Wang Y, Zhou S, Luo S, Lin Y, Wang R, Jia Q, Feng T, Zhao G, Yin X, Yang F (2025). Unveiling the components, physiological functions and emerging trends of hydroxycinnamic acid amides in goji berry (Lycium). Food Res Int Ott Ont.

[R90] Weinreb RN, Aung T, Medeiros FA (2014). The pathophysiology and treatment of glaucoma: a review. JAMA.

[R91] Winkler PA, Occelli LM, Petersen-Jones SM (2020). Large animal models of inherited retinal degenerations: a review. Cells.

[R92] Wong WL, Su X, Li X, Cheung CMG, Klein R, Cheng CY, Wong TY (2014). Global prevalence of age-related macular degeneration and disease burden projection for 2020 and 2040: a systematic review and meta-analysis. Lancet Glob Health.

[R93] Wu J, Zhang J, Lin B (2025). Dual-targeting CSF1R signaling attenuates neurotoxic myeloid activation and preserves photoreceptors in retinitis pigmentosa. J Neuroinflammation.

[R94] Yang D, So KF, Lo AC (2017). Lycium barbarum polysaccharide extracts preserve retinal function and attenuate inner retinal neuronal damage in a mouse model of transient retinal ischaemia. Clin Experiment Ophthalmol.

[R95] Yang L, Li Z, Fang J (2024). Scutellarin alleviates diabetic retinopathy via the suppression of nucleotide-binding oligomerization domain (NOD)-like receptor pyrin domain containing protein 3 inflammasome activation. Curr Eye Res.

[R96] Yang Y, Li W, Li Y, Wang Q, Gao L, Zhao J (2014). Dietary Lycium barbarum polysaccharide induces Nrf2/ARE pathway and ameliorates insulin resistance induced by high-fat via activation of PI3K/AKT signaling. Oxid Med Cell Longev.

[R97] Yang YJ, Wang Y, Deng Y, Liu XQ, Lu J, Peng J, Li J, Zhou YS, Zhu HA, Li B, Qin YH, Peng QH (2023). Lycium barbarum polysaccharides regulating miR-181/Bcl-2 decreased autophagy of retinal pigment epithelium with oxidative stress. Oxid Med Cell Longev.

[R98] Yao F, Zhang X, Yao X, Ren X, Xia X, Jiang J, Ding L (2021). Peroxisome proliferator-activated receptor α activation protects retinal ganglion cells in ischemia-reperfusion retinas. Front Med.

[R99] Yao M, Zhang L, Wang L (2023). Astragaloside IV: a promising natural neuroprotective agent for neurological disorders. Biomed Pharmacother Biomedecine Pharmacother.

[R100] Yao Q, Yang Y, Lu X, Zhang Q, Luo M, Li PA, Pan Y (2018). Lycium barbarum polysaccharides improve retinopathy in diabetic Sprague-Dawley rats. Evid-Based Complement Altern Med.

[R101] Yao Q, Zhou Y, Yang Y, Cai L, Xu L, Han X, Guo Y, Li PA (2020). Activation of Sirtuin1 by lyceum barbarum polysaccharides in protection against diabetic cataract. J Ethnopharmacol.

[R102] Yau JWY (2012). Global prevalence and major risk factors of diabetic retinopathy. Diabetes Care.

[R103] Ye H, Feng Y, Xiang W, Lin Z, Li Y, Hu W, Liu K, Tao S, Shu Q, Wang J, Xu F, Xu Y, Wei Y, Huang J (2025). Ferroptosis contributes to retinal ganglion cell loss in GLAST knockout mouse model of normal tension glaucoma. Invest Ophthalmol Vis Sci.

[R104] Zhou B, Xia H, Yang L, Wang S, Sun G (2022). The effect of lyciumbarbarum polysaccharide on the glucose and lipid metabolism: a systematic review and meta-analysis. J Am Nutr Assoc.

[R105] Zhu Y, Zhao Q, Gao H, Peng X, Wen Y, Dai G (2016). Lycium barbarum polysaccharides attenuates N-methy-N-nitrosourea-induced photoreceptor cell apoptosis in rats through regulation of poly (ADP-ribose) polymerase and caspase expression. J Ethnopharmacol.

